# Supplementary material for: How are trial outcomes prioritised by stakeholders from different regions? Analysis of an international Delphi survey to develop a core outcome set in gastric cancer surgery
Source: PLoS One. 2021 Dec 31;16(12):e0261937. doi: 10.1371/journal.pone.0261937 (PMC8719722; doi:10.1371/journal.pone.0261937)
Supplement: Attachment — Submitted filename: Response to reviewer comments 011121.docx [file pone.0261937.s002.docx]

Funded by:

1^st^ of November 2021

Dear Editor

**Revision of manuscript PONE-D-20-40628**

We would like to thank the reviewers for taking the time to provide their valuable comments which have been carefully considered. Where necessary, the manuscript has been updated to reflect the recommendations made and we have provided detailed responses below.

As you have requested, there are no data sharing limitations, and a minimum dataset has been provided with the submission and is included in the tables.

Yours sincerely

Bilal Alkhaffaf, PhD, FRCS

**Consultant Oesophago-Gastric Surgeon, Salford NHS Foundation Trust, UK**
**Honorary Senior Lecturer, University of Manchester, UK**

**Responses to Editor’s Comments**

Overall the manuscript is very interesting as it investigates an underappreciated topic in surgical research. In this regard, authors should consider that most of readers are not familiar with the study topic and need some more guidance through the manuscript.

This is particularly important as the manuscript is well written, study hypothesis, aims and methodology are clear and conclusions robust. Also, the Discussion section stated “Ultimately, a COS will only achieve its stated goals if researchers use it.”thus supporting the dissemination of the results of this study. Overall, I would request authors to improve their study presentation in order to make it more readable.

1. **Title is difficult to get and there are repetitions. I would consider a simpler title such as “Development of a core outcome set for clinical trials in gastric cancer surgery: the GASTROS study”.**

This manuscript aims specifically to explore how outcomes are prioritised by stakeholders in different regions. If outcomes are valued differently, then achieving a balance when recruiting participants for the consensus process becomes more important in reducing bias. The study is not the description of the final COS which has been published elsewhere (<https://academic.oup.com/bjs/advance-article/doi/10.1093/bjs/znab192/6308782?searchresult=1>). As requested, the title has been simplified to reflect this:

‘How are trial outcomes prioritised by stakeholders from different regions? Analysis of an international Delphi survey to develop a core outcome set in gastric cancer surgery.’

2. **Abstract is not very informative of study findings. The result section should include the outcomes to be prioritized and report overall rates, otherwise it is very difficult to have an idea of what the manuscript analysed.**

There were 56 outcomes to prioritise. This has now been added to the methods section. The outcomes on which consensus was reached to include in the COS have been added to the abstract. For brevity, those which were excluded or where no consensus was reached have not been added to the abstract but can still be found in the main manuscript.

3. **Result section is hard to get. For instance, outcomes should be grouped according to their main area, for instance: prognosis, post-operative morbidity, quality of life, long-term morbidity. This may make it easier for Authors to provide a more narrative reporting of their result section, which currently is just a reminder to tables, which is hard to follow for readers. Again , please consider taking the reader through the study results rather than ask him/her to look at tables.**

As requested, the results section has been expanded and a narrative description of the results has now been incorporated. A taxonomy used to organise outcomes for Delphi participants has been cited and tables now reflect these ‘core outcome areas’ as recommended by the reviewer.

4. **Clearly authors want to reach surgeons and oncologists with this paper. How they plan to reach nurses? What about patients? It would be interesting to have authors’ strategy reported in the discussion.**

A statement regarding our dissemination policy has now been added to the discussion section. Updating all stakeholder groups involved in this study is certainly an important consideration which was set out *a priori*.

5. **A study limitation is the lack of medical oncologists and radiation oncologists. Can you please explain reasons for considering only surgeons for a disease that is treated multidisciplinary? This issue should be discussed.**

The scope of the study and the reasons why other healthcare providers were not invited to participate in the study has now been added to the limitations section of the discussion. The justification for this has been included in the methods section.

6. **In the study conclusion it should be reported that three stakeholders (surgeons, nurses and patients) were considered. There should at last be some examples of included outcomes, for instance those with the highest agreement.**

The conclusion has been modified to reflect the recommendation to explicitly describe the stakeholders invited to participate in the study. The outcomes where consensus agreement was reached amongst all stakeholders has also been included.

7. **I would appreciate some comment on the generalizability of these findings to other surgical oncology disease. Authors comment that gastric cancer has extensive differences compared to breast cancer. However these findings may be valuable, at least to some extents, for GI cancers. Please comment.**

As this is the first time that a group has examined regional variation as a possible variable on how outcomes are prioritised, it is difficult to be able to comment on whether the findings from this study will be applicable to other COS studies – surgical oncology or otherwise. We certainly believe that this requires some further study and we have added a section into the discussion which encourages other developers to collect this type of baseline data during the registration period of the survey.

The differences that we discuss between gastric and breast cancer relate to the way in which stakeholder groups were assembled during the respective COS, and not regarding the disease itself. Further work is already underway to identify commonalities and differences between core outcome sets in surgical oncology.

8. **There is a typo in the first line of 4.4 – “is was”.**

This mistake has been corrected.

**Response to Reviewer:**

This is an interesting paper . I admire your efforts at recruitment for this Delphi. In answer to the journal’s specific questions:
• Further data/evidence of analysis is offered in response to personal request.
• The manuscript is parsimonious, and well written and presented.
• It offers the tabular analysis I would expect to see to support the body of the paper, and the conclusions drawn seem sound in relation to the data presented. The sample sizes (you do point this out) are too small to make definitive conclusions, and there are many confounding factors (you also point this out) that may play a role in the variation you report.

As a reader:
I was initially not persuaded that subgroup analysis would be pragmatic in this context, but I inevitably felt drawn in by the tables and percentages, and was struck by Table 5 and how the results hang together here. You do state the key point that a COS is intended to allow synthesis, and to be useful in international trials – reminding the reader that the heart of COS methodology is the search for consensus rather than divergence. So the intention to analyse subgroups did raise some questions, but I think I’m persuaded that this was worth exploring (it made me think, always a good sign). Generally speaking, I felt that the paper was carefully worded so as not to stray into too much speculation.

Issues that came to mind as I read through (these are very general):

1. **Section 2.3 Data analysis and interpretation - perhaps I'm misinterpreting this, but the first sentence suggests that participants included in the analysis did not complete the whole Delphi survey - in fact, some of them only completed 50% of the survey? It seemed to me that including surveys that were only partially filled in meant that you are not comparing like-with-like across the different outcomes, but I may be missing something here.**

The study management group considered whether to include partially completed surveys. The argument against excluding partially completed surveys related to the possibility that valid responses would be excluded. Ultimately, we settled on including surveys which had been at least 50% completed as it was probable that these participants had understood the purpose of the study. Furthermore, some participants who had not fully completed the first Delphi went on to complete the second survey which supported our choice. The number of partially completed studies was small (3%) and most partially completed surveys had prioritised closer to 100% of outcomes rather than the 50% minimum response threshold that was set.

2. **I would have appreciated a sentence or two summarising the concrete differences in pathology, treatment and outcome between regions (rather than the statement that there are differences), so that I could do some independent thinking around this.**

As requested, specific examples of regional differences have now been added to the introduction.

3. **I don’t see why this paper shouldn’t flag up some potential issues around the consensus approach (rather than just reassuring us!), given that the same methodology for COS development is used across a variety of subject areas which are very different in shape. The COS development process is influenced by the nature of the condition being explored, whether you are trying to tie down outcomes for a single intervention or a range of interventions for the same condition, and the breadth of stakeholder roles involved (which is inevitably affected by the two previous issues). I wanted just a little more information about the background so that I could think about these issues without having to look them up in a separate paper.**

A section which discusses the merits and limitations of using this methodological approach has now been added to the last paragraph before the conclusion.

4. **It’s a benefit to this analysis to have a low number of stakeholder groups, and it’s a given that a study steering group should carefully think about which role perspectives (besides patients) they should include as respondents in a Delphi survey. I felt that the call to consider more finely grained characteristics of potential respondents when you plan recruitment was perhaps optimistic, as this is partly resource-dependent, and capacity in research institutions varies greatly. Not all COS projects would have funding to access demographic information about patients in order to guide recruitment (and adding demographic questions to the survey could be a burden and increase attrition). I’m not saying this isn’t a good idea for recruitment – just in an ideal world. I wonder whether the paper should touch on capacity issues and make some clear calls for investment in this kind of approach for COS studies that are likely to benefit from it, which would in turn feed back to funders of COS projects?**

The reviewer has made an extremely important point about limited resources often available during COS during studies. However, the demographic information that was gathered at the start of the Delphi survey was relatively basic, was self-reported by participants and did not take long to complete. The concern regarding the burden of supplying demographic details did not materialise during our study which achieved a retention rate of 70% between round 1 and round 2. This has been added to the section 4.1 in the discussion. A call and justification to ensure appropriate funding for COS studies has been incorporated in the discussion.

**Journal Requirements**

1. **Please ensure that your manuscript meets PLOS ONE's style requirements, including those for file naming. The PLOS ONE style templates can be found at**
   [**https://journals.plos.org/plosone/s/file?id=wjVg/PLOSOne_formatting_sample_main_body.pdf**](http://smtp2.srft.nhs.uk:32224/?dmVyPTEuMDAxJiY1ODk0MDJjOGE4NWIxOGFjYT02MEREMDAzRF85NDcwMV8xMjI2Nl8xJiY1ODg2YmRhOGM4NThmNjk9MTIyMiYmdXJsPWh0dHBzJTNBJTJGJTJGam91cm5hbHMlMkVwbG9zJTJFb3JnJTJGcGxvc29uZSUyRnMlMkZmaWxlJTNGaWQlM0R3alZnJTJGUExPU09uZSU1RmZvcm1hdHRpbmclNUZzYW1wbGUlNUZtYWluJTVGYm9keSUyRXBkZg==) **and** 
   [**https://journals.plos.org/plosone/s/file?id=ba62/PLOSOne_formatting_sample_title_authors_affiliations.pdf**](http://smtp2.srft.nhs.uk:32224/?dmVyPTEuMDAxJiY1ODk0MDJjOGE4NWIxOGFjYT02MEREMDAzRF85NDcwMV8xMjI2Nl8xJiY1ODg2YmRhOGM4NThmNjk9MTIyMiYmdXJsPWh0dHBzJTNBJTJGJTJGam91cm5hbHMlMkVwbG9zJTJFb3JnJTJGcGxvc29uZSUyRnMlMkZmaWxlJTNGaWQlM0RiYTYyJTJGUExPU09uZSU1RmZvcm1hdHRpbmclNUZzYW1wbGUlNUZ0aXRsZSU1RmF1dGhvcnMlNUZhZmZpbGlhdGlvbnMlMkVwZGY=)

The revised manuscript has been formatted according to journal’s style requirements.

2. **Please review your reference list to ensure that it is complete and correct. If you have cited papers that have been retracted, please include the rationale for doing so in the manuscript text, or remove these references and replace them with relevant current references. Any changes to the reference list should be mentioned in the rebuttal letter that accompanies your revised manuscript. If you need to cite a retracted article, indicate the article’s retracted status in the References list and also include a citation and full reference for the retraction notice.**

This does not apply to our study.

3. **Thank you for including your ethics statement:  "The study was given ethical approval by the North West - Greater Manchester East Research Ethics Committee (18/NW/0347) and governance approvals by Manchester University Hospitals NHS Foundation Trust.".
   Please provide additional details regarding participant consent. In the ethics statement in the Methods and online submission information, please ensure that you have specified (1) whether consent was informed and (2) what type you obtained (for instance, written or verbal, and if verbal, how it was documented and witnessed). 

   Once you have amended this/these statement(s) in the Methods section of the manuscript, please add the same text to the “Ethics Statement” field of the submission form (via “Edit Submission”).

   For additional information about PLOS ONE ethical requirements for human subjects research, please refer to** [**http://journals.plos.org/plosone/s/submission-guidelines#loc-human-subjects-research.**](http://smtp2.srft.nhs.uk:32224/?dmVyPTEuMDAxJiYwNjg4MDFkNGY1MWEwNWI5ZT02MEREMDAzRF85NDcwMV8xMjI2Nl8xJiZkZDc3N2Q5OTA5OThlNjI9MTIyMiYmdXJsPWh0dHAlM0ElMkYlMkZqb3VybmFscyUyRXBsb3MlMkVvcmclMkZwbG9zb25lJTJGcyUyRnN1Ym1pc3Npb24tZ3VpZGVsaW5lcyUyM2xvYy1odW1hbi1zdWJqZWN0cy1yZXNlYXJjaCUyRSUyNm5ic3AlM0I=)

The ethics statement has been updated as requested.

4. **Please include additional information regarding the survey or questionnaire used in the study and ensure that you have provided sufficient details that others could replicate the analyses. For instance, if you developed a questionnaire as part of this study and it is not under a copyright more restrictive than CC-BY, please include a copy, in both the original language and English, as Supporting Information.**

A copy of the survey has now been included as a supplementary file. This is not bound by copyright restrictions.

5. **In your Methods section, please provide additional information about the participant recruitment method and the demographic details of your participants. Please ensure you have provided sufficient details to replicate the analyses such as:
   - the recruitment date range (month and year)
   - a description of any inclusion/exclusion criteria that were applied to participant recruitment
   - a table of relevant demographic details
   - a statement as to whether your sample can be considered representative of a larger population
   - a description of how participants were recruited.**

- The recruitment dates have been added to the first paragraph in the methods section.
- Eligibility criteria is included in section 2.1
- Table 1 summarises the demographic details
- Statement regarding the representative nature of our sample has been included in the first paragraph of the discussion.
- Recruitment summary has now been added to section 2.1

6. **Please list the name and version of any software package used for statistical analysis, alongside any relevant references. For more information on PLOS ONE's expectations for statistical reporting, please see** [**https://journals.plos.org/plosone/s/submission-guidelines.#loc-statistical-reporting.**](http://smtp2.srft.nhs.uk:32224/?dmVyPTEuMDAxJiY1ODk0MDJjOGE4NWIxOGFjYT02MEREMDAzRF85NDcwMV8xMjI2Nl8xJiY1ODg2YmRhOGM4NThmNjk9MTIyMiYmdXJsPWh0dHBzJTNBJTJGJTJGam91cm5hbHMlMkVwbG9zJTJFb3JnJTJGcGxvc29uZSUyRnMlMkZzdWJtaXNzaW9uLWd1aWRlbGluZXMlMkUlMjNsb2Mtc3RhdGlzdGljYWwtcmVwb3J0aW5nJTJF)

SPSS has been cited in section 2.3

7. **We note that the grant information you provided in the ‘Funding Information’ and ‘Financial Disclosure’ sections do not match.**

**When you resubmit, please ensure that you provide the correct grant numbers for the awards you received for your study in the ‘Funding Information’ section.**

JMB and PRW’s funding was not used for this study. The two sections now match.

8. **We note that you have indicated that data from this study are available upon request. PLOS only allows data to be available upon request if there are legal or ethical restrictions on sharing data publicly. For more information on unacceptable data access restrictions, please see** [**http://journals.plos.org/plosone/s/data-availability#loc-unacceptable-data-access-restrictions.**](http://smtp2.srft.nhs.uk:32224/?dmVyPTEuMDAxJiYwNjg4MDFkNGY1MWEwNWI5ZT02MEREMDAzRF85NDcwMV8xMjI2Nl8xJiZkZDc3N2Q5OTA5OThlNjI9MTIyMiYmdXJsPWh0dHAlM0ElMkYlMkZqb3VybmFscyUyRXBsb3MlMkVvcmclMkZwbG9zb25lJTJGcyUyRmRhdGEtYXZhaWxhYmlsaXR5JTIzbG9jLXVuYWNjZXB0YWJsZS1kYXRhLWFjY2Vzcy1yZXN0cmljdGlvbnMlMkUlMjZuYnNwJTNC)

**In your revised cover letter, please address the following prompts:**

**a) If there are ethical or legal restrictions on sharing a de-identified data set, please explain them in detail (e.g., data contain potentially sensitive information, data are owned by a third-party organization, etc.) and who has imposed them (e.g., an ethics committee). Please also provide contact information for a data access committee, ethics committee, or other institutional body to which data requests may be sent.**

**b) If there are no restrictions, please upload the minimal anonymized data set necessary to replicate your study findings as either Supporting Information files or to a stable, public repository and provide us with the relevant URLs, DOIs, or accession numbers. For a list of acceptable repositories, please see** [**http://journals.plos.org/plosone/s/data-availability#loc-recommended-repositories.**](http://smtp2.srft.nhs.uk:32224/?dmVyPTEuMDAxJiYwNjg4MDFkNGY1MWEwNWI5ZT02MEREMDAzRF85NDcwMV8xMjI2Nl8xJiZkZDc3N2Q5OTA5OThlNjI9MTIyMiYmdXJsPWh0dHAlM0ElMkYlMkZqb3VybmFscyUyRXBsb3MlMkVvcmclMkZwbG9zb25lJTJGcyUyRmRhdGEtYXZhaWxhYmlsaXR5JTIzbG9jLXJlY29tbWVuZGVkLXJlcG9zaXRvcmllcyUyRQ==)

**We will update your Data Availability statement on your behalf to reflect the information you provide.**

Minimum data has already been included in the tables as requested. Data availability statement has been changed.

9. [**One**](http://smtp2.srft.nhs.uk:32224/?dmVyPTEuMDAxJiYxZTkwMWFkN2UxNWE1OGU3ZT02MEREMDAzRF85NDcwMV8xMjI2Nl8xJiZjZTg3NWQzOGI4Mjk0Nzc9MTIyMiYmdXJsPWh0dHAlM0ElMkYlMkY5JTJFT25l) **of the noted authors is a group or consortium GASTROS International Working Group . In addition to naming the author group, please list the individual authors and affiliations within this group in the acknowledgments section of your manuscript. Please also indicate clearly a lead author for this group along with a contact email address.**

This has been done on the final page of the submission.

10. **We note that Figure 1 in your submission contain map image which may be copyrighted. All PLOS content is published under the Creative Commons Attribution License (CC BY 4.0), which means that the manuscript, images, and Supporting Information files will be freely available online, and any third party is permitted to access, download, copy, distribute, and use these materials in any way, even commercially, with proper attribution. For these reasons, we cannot publish previously copyrighted maps or satellite images created using proprietary data, such as Google software (Google Maps, Street View, and Earth). For more information, see our copyright guidelines:** [**http://journals.plos.org/plosone/s/licenses-and-copyright.**](http://smtp2.srft.nhs.uk:32224/?dmVyPTEuMDAxJiYwNjg4MDFkNGY1MWEwNWI5ZT02MEREMDAzRF85NDcwMV8xMjI2Nl8xJiZkZDc3N2Q5OTA5OThlNjI9MTIyMiYmdXJsPWh0dHAlM0ElMkYlMkZqb3VybmFscyUyRXBsb3MlMkVvcmclMkZwbG9zb25lJTJGcyUyRmxpY2Vuc2VzLWFuZC1jb3B5cmlnaHQlMkU=)
     
    **We require you to either (1) present written permission from the copyright holder to publish these figures specifically under the CC BY 4.0 license, or (2) remove the figures from your submission:
     
    a. You may seek permission from the original copyright holder of Figure 1 to publish the content specifically under the CC BY 4.0 license.  
     
    We recommend that you contact the original copyright holder with the Content Permission Form (**[**http://journals.plos.org/plosone/s/file?id=7c09/content-permission-form.pdf**](http://journals.plos.org/plosone/s/file?id=7c09/content-permission-form.pdf)**) and the following text:
    “I request permission for the open-access journal PLOS ONE to publish XXX under the Creative Commons Attribution License (CCAL) CC BY 4.0 (**[**http://creativecommons.org/licenses/by/4.0/**](http://creativecommons.org/licenses/by/4.0/)**). Please be aware that this license allows unrestricted use and distribution, even commercially, by third parties. Please reply and provide explicit written permission to publish XXX under a CC BY license and complete the attached form.”
     
    Please upload the completed Content Permission Form or other proof of granted permissions as an "Other" file with your submission.
     
    In the figure caption of the copyrighted figure, please include the following text: “Reprinted from [ref] under a CC BY license, with permission from [name of publisher], original copyright [original copyright year].”
     
    b. If you are unable to obtain permission from the original copyright holder to publish these figures under the CC BY 4.0 license or if the copyright holder’s requirements are incompatible with the CC BY 4.0 license, please either i) remove the figure or ii) supply a replacement figure that complies with the CC BY 4.0 license. Please check copyright information on all replacement figures and update the figure caption with source information. If applicable, please specify in the figure caption text when a figure is similar but not identical to the original image and is therefore for illustrative purposes only.
    The following resources for replacing copyrighted map figures may be helpful:
     
    USGS National Map Viewer (public domain):** [**http://viewer.nationalmap.gov/viewer/**](http://smtp2.srft.nhs.uk:32224/?dmVyPTEuMDAxJiYwMjhkMDFjOWJhMTkxYWJmYj02MEREMDAzRF85NDcwMV8xMjI2Nl8xJiYyODk3Y2Q5OTVkOTk2NmU9MTIyMiYmdXJsPWh0dHAlM0ElMkYlMkZ2aWV3ZXIlMkVuYXRpb25hbG1hcCUyRWdvdiUyRnZpZXdlciUyRg==)
    **The Gateway to Astronaut Photography of Earth (public domain):** [**http://eol.jsc.nasa.gov/sseop/clickmap/**](http://smtp2.srft.nhs.uk:32224/?dmVyPTEuMDAxJiYxNzk3MGY4OWJjMWEwMWYxYj02MEREMDAzRF85NDcwMV8xMjI2Nl8xJiYxZDQ3ZWQ5OTNkOTgzNmI9MTIyMiYmdXJsPWh0dHAlM0ElMkYlMkZlb2wlMkVqc2MlMkVuYXNhJTJFZ292JTJGc3Nlb3AlMkZjbGlja21hcCUyRg==)
    **Maps at the CIA (public domain):** [**https://www.cia.gov/library/publications/the-world-factbook/index.html**](http://smtp2.srft.nhs.uk:32224/?dmVyPTEuMDAxJiYxMThiMTg4OGI3MWMxNWFjYT02MEREMDAzRF85NDcwMV8xMjI2Nl8xJiYzZDU2Mjk5OTM4MzgyNmI9MTIyMiYmdXJsPWh0dHBzJTNBJTJGJTJGd3d3JTJFY2lhJTJFZ292JTJGbGlicmFyeSUyRnB1YmxpY2F0aW9ucyUyRnRoZS13b3JsZC1mYWN0Ym9vayUyRmluZGV4JTJFaHRtbA==) **and** [**https://www.cia.gov/library/publications/cia-maps-publications/index.html**](http://smtp2.srft.nhs.uk:32224/?dmVyPTEuMDAxJiYxMThiMTg4OGI3MWMxNWFjYT02MEREMDAzRF85NDcwMV8xMjI2Nl8xJiYzZDU2Mjk5OTM4MzgyNmI9MTIyMiYmdXJsPWh0dHBzJTNBJTJGJTJGd3d3JTJFY2lhJTJFZ292JTJGbGlicmFyeSUyRnB1YmxpY2F0aW9ucyUyRmNpYS1tYXBzLXB1YmxpY2F0aW9ucyUyRmluZGV4JTJFaHRtbA==)
    **NASA Earth Observatory (public domain):** [**http://earthobservatory.nasa.gov/**](http://smtp2.srft.nhs.uk:32224/?dmVyPTEuMDAxJiYwNDkyMGZkM2I0MDcwZWYwYT02MEREMDAzRF85NDcwMV8xMjI2Nl8xJiZjYzY2OGQ3Y2Q5MThmNzE9MTIyMiYmdXJsPWh0dHAlM0ElMkYlMkZlYXJ0aG9ic2VydmF0b3J5JTJFbmFzYSUyRWdvdiUyRg==)
    **Landsat:** [**http://landsat.visibleearth.nasa.gov/**](http://smtp2.srft.nhs.uk:32224/?dmVyPTEuMDAxJiYxZjk3MDdjNWI3MTAxMmJmYj02MEREMDAzRF85NDcwMV8xMjI2Nl8xJiYwZDM3Mzk4OGQ5NzkzNjY9MTIyMiYmdXJsPWh0dHAlM0ElMkYlMkZsYW5kc2F0JTJFdmlzaWJsZWVhcnRoJTJFbmFzYSUyRWdvdiUyRg==)
    **USGS EROS (Earth Resources Observatory and Science (EROS) Center) (public domain):** [**http://eros.usgs.gov/#**](http://smtp2.srft.nhs.uk:32224/?dmVyPTEuMDAxJiYxZTkwMWFkN2UxNWE1OGJiYj02MEREMDAzRF85NDcwMV8xMjI2Nl8xJiYwYzg2ODk4OTY4NTg3NzQ9MTIyMiYmdXJsPWh0dHAlM0ElMkYlMkZlcm9zJTJFdXNncyUyRWdvdiUyRiUyMw==)
    **Natural Earth (public domain):** [**http://www.naturalearthdata.com/**](http://smtp2.srft.nhs.uk:32224/?dmVyPTEuMDAxJiYxNzg4MGJjNmE5MDExZmJhYT02MEREMDAzRF85NDcwMV8xMjI2Nl8xJiYzZDM3YTk4ODA5OThkMjg9MTIyMiYmdXJsPWh0dHAlM0ElMkYlMkZ3d3clMkVuYXR1cmFsZWFydGhkYXRhJTJFY29tJTJG)

This image was created by the team and contains no copyrighted material.

9. **Please include a copy of Table 1 which you refer to in your text on page 18.**

This is included in the manuscript.

10. **We note you have included a table to which you do not refer in the text of your manuscript. Please ensure that you refer to Table 31 and 32 in your text; if accepted, production will need this reference to link the reader to the Table.**

Please can you expand on your comment? I cannot find reference to tables 31 or 32 in the text.
